# Tectorigenin inhibits inflammatory responses in murine inflammatory bowel disease and LPS‐stimulated macrophages via inactivating MAPK signaling pathway

**DOI:** 10.1002/iid3.1077

**Published:** 2024-05-09

**Authors:** Hong Huang, Sanhui Tang, Yanghong Zhou, Yi Cai

**Affiliations:** ^1^ Department of TCM and Integrated TCM and Western Medicine Hunan Provincial People's Hospital, The First Affiliated Hospital of Hunan Normal University Changsha China; ^2^ Department of Liver and Gall Surgical Hunan Provincial People's Hospital, The First Affiliated Hospital of Hunan Normal University Changsha China

**Keywords:** IBD, LPS, macrophages, MAPK signaling pathway, tectorigenin

## Abstract

**Background:**

Considering the antihepatitis effects of Tectorigenin (TEC), and the same adenosine mitogen‐activated protein kinase (MAPK) pathway in both hepatitis and inflammatory bowel disease (IBD) models, exploring the role of TEC in IBD is contributive to develop a new treatment strategy against IBD.

**Methods:**

The IBD mouse model was constructed by feeding with dextran sodium sulfate (DSS) and injection of TEC. Afterward, the mouse body weight, colon length, and disease activity index (DAI) were tested to assess the enteritis level. Mouse intestine lesions were detected by hematoxylin and eosin staining. Murine macrophages underwent lipopolysaccharide (LPS) induction to establish an inflammation model. Cell viability was determined by cell counting kit‐8 assay. Enzyme‐linked immunosorbent assay was performed to measure interleukin 6 (IL‐6) and tumor necrosis factor‐α (TNF‐α) levels. Cyclooxygenase‐2 (COX‐2) and inducible nitric oxide synthase (iNOS) expressions were quantified via quantitative reverse transcription polymerase chain reaction. Levels of MAPK pathway‐related proteins (p‐P38, P38, p‐Jun N‐terminal kinase (JNK), JNK, signal‐regulated kinase (ERK), p‐ERK), COX‐2 and iNOS were quantitated by Western blot.

**Results:**

TEC improved the inflammatory response through ameliorating weight loss, shortening colon, and increasing DAI score in IBD mouse. Expressions of intestinal inflammatory factors (IL‐6, TNF‐α, iNOS and COX‐2) and MAPK pathway‐related proteins (p‐P38, p‐JNK, and p‐ERK) were increased both in DSS‐induced mouse intestinal tissue, but TEC inhibited expressions of inflammatory factors. The same increased trend was identified in LPS‐induced macrophages, but TEC improved macrophage inflammation, as evidenced by downregulation of inflammatory factors.

**Conclusion:**

TEC mitigates IBD and LPS‐induced macrophage inflammation in mice via inhibiting MAPK signaling pathway.

## Graphical abstract

(1) Inflammatory bowel disease (IBD) model was established by dextran sodium sulfate‐induced mice. (2) Tectorigenin improves enteritis damage by inhibiting mitogen‐activated protein kinase pathway in IBD mice. (3) Lipopolysaccharide (LPS)‐induced inflammation in macrophages. (4) Tectorigenin inhibited inflammation in LPS‐induced macrophages.

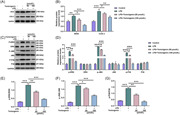

## 1 INTRODUCTION

Inflammatory bowel disease (IBD) is a chronic nonspecific inflammatory disease that occurs in the gastrointestinal tract, comprising two major disorders ulcerative colitis (UC) and Crohn's disease.^1^ The clinical symptoms of IBD involve weight loss, recurrent diarrhea, abdominal pain, hematochezia, fistula lesion, perianal disease, and so on. Whitney et al.^2^ IBD has a high recurrence rate and is accompanied with many complications, such as intestinal fibrosis, colorectal cancer, and anemia, which are life‐threatening.^3^ Today, the incidence of IBD continues to rise, which challenges the global public health.^4^ The mechanism of the induction and occurrence of IBD has not been deciphered yet, and the treatment mainly depends on anti‐inflammatory drugs and immunomodulators.^5^, ^6^ However, the clinical application of these drugs is severely restricted due to the side effects. Remarkably, traditional Chinese medicine has received increasing attention in the treatment of IBD because of its anti‐inflammatory effects and high recognition by patients.^7^

Tectorigenin (TEC) is isolated from the traditional Chinese medicine *Belamcanda chinensis*,^8^ with antiproliferative, antioxidant, and anti‐inflammatory properties.^9^ Reportedly, in dextran sodium sulfate (DSS)‐induced mouse hepatitis models, TEC attenuates the inflammatory response via downregulating toll‐like receptor 4 (TLR4) expression, blocking mitogen‐activated protein kinase (MAPK) and nuclear factor‐κB pathway, and boosting lipopolysaccharide (LPS)‐induced autophagy of macrophages.^10^ Hepatitis model is similar to IBD model in terms of MAPK inflammatory pathway.^11^, ^12^, ^13^, ^14^ Therefore, this study hypothesized that TEC may also have anti‐inflammatory effects in IBD.

Macrophage is a main immune cells, playing a role in activating, maintaining and attenuating inflammation.^15^ LPS‐activated macrophages (IFN‐γ) induce innate immune responses and produce proinflammatory mediators that lead to inflammation, so therapeutic interventions targeted macrophage may serve as a potential method to suppress inflammation.^15^ Besides, inhibiting macrophages can effectively improve UC in mice.^16^ In the hepatitis model, TEC inhibits LPS‐induced inflammation by promoting macrophage autophagy.^17^ Accordingly, we further speculated that TEC may also attenuate the inflammation of LPS‐induced macrophages.

In view of the above information, we established an IBD mouse model and LPS‐induced macrophage model to explore the effect of TEC on IBD, in an effort to provide a new drug for treating IBD and reduce the pain suffered by patients. Moreover, we hope to provide a reference for treatment of other diseases with a same inflammatory pathway from a molecular perspective, and make a contribution to public health.

## 2 MATERIALS AND METHODS

### 2.1 C57BL/6 mouse model of IBD

In this study, male C57BL/6 mice (*n* = 30, 10 mice/group, weight: 22–28 g, age: >6 weeks; J006, junke biological Co., Ltd.) were kept in a separate cage under normal laboratory conditions (temperature of 24 ± 2°C, air humidity of 50 ± 15%, and light‐dark cycle for 12 h). These mice can drink freely and receive a normal pellet diet. After a week of acclimation, 30 mice were randomly assigned to three groups.

Control group: The mice can eat and drink normally, and were injected intraperitoneally with phosphate‐buffered saline (PBS, C0221A, Beyotime) containing 5% dimethyl sulfoxide (D2650, MillipoRe SiGMa) for 7 days.

Model group: 5% (w/v) DSS (21655, Thermo Fisher Scientific) was added to drinking water for 7 days to induce acute colitis in mice.^16^ On the 8th day, sterile normal drinking water was provided. While modeling, mice were injected intraperitoneally with PBS containing 5% dimethyl sulfoxide in the same amount as control group for 7 days.

TEC group: Acute colitis was induced in mice through drinking water with 5% (w/v) DSS for 7 days, and then sterilized normal drinking water was provided on the 8th day. While modeling, mice were injected with TEC (25 mg/kg) dissolved in PBS containing 5% dimethyl sulfoxide into the abdominal cavity for 7 days.

On the 9th day, all mice were killed under anesthetization using 1% pentobarbital sodium (676‐50, Sagent Pharmaceuticals) at a dose of 50 mg/kg, and the intestinal tissues were collected and stored for later use.

### 2.2 Treatment of macrophages

RAW264.7 murine macrophages, purchased from American Type Culture Collection (ATCC, CRL‐2471), were cultured in Dulbecco's modified Eagle medium (DMEM, 30‐2002, ATCC) supplemented with 10% fatal bovine serum (30‐2020, ATCC) at 37°C under 5% CO_2_. After that, the cells were treated with different concentrations of TEC (0, 1, 10, 50, 100, 200 μM) for 24 h.^17^ Later, cells were assigned into four groups: control group (cells without treatment), LPS group (cells treated with LPS (1 μg/mL) for 24 h), as well as TEC (50) and TEC (100) groups (cells firstly treated with 50 μM and 100 μM TEC, and then with LPS (1 μg/mL) for 24 h).^17^

### 2.3 Evaluation of IBD

For the first 7 days of the experiment, the weight of mice was measured. At the same time, the hematochezia and diarrhea of the mice were observed and recorded to score the disease activity index (DAI). Table 1 displayed the DAI scoring criteria.

**Table 1:** Scoring criteria for the disease activity index.

Score	Weight loss (%)	Diarrhea	Hematochezia
0	5	No	No
1	5–10	‐	‐
2	10–15	Soft but formed stool	Hematochezia visible to the naked eye
3	15–20	‐	‐
4	>20	Watery stool	Rectal bleeding

After the experiment, the mice were killed on the 9th day, followed by collection of the intestines, as well as measurement and analysis of the colon length.

### 2.4 Hematoxylin and eosin (HE) staining

The intestinal tissue was fixed in 10% formalin buffer and paraffined after dehydration. The tissue was sliced by an automatic microtome into sections with a thickness of 5 µm and stained with HE reagent (C0105, Beyotime). The sections were fixed for more than 10 min with the fixative, and washed with distilled water for 2 min. Then, the sections were stained with hematoxylin staining solution for 5–10 min and immersed in tap water to rinse off excess staining solution for about 10 min. After being washed again with distilled water (a few seconds), differentiated by the differentiation solution for about 2–30 s and rinsed with tap water for 10 min, the sections were colored by eosin solution for 1 min. Later, washing with 70% ethanol for 10 s, 80% ethanol for 10 s, 90% ethanol for 10 s, and absolute ethanol for 10 s was performed. Next, the sections were transparentized with xylene for 5 min twice, and mounted with neutral gum. Finally, the stained sections were photographed under a microscope (Leica Microsystems) at a magnification of 200×. The histopathological scoring of the colon was performed according to the followed histopathological changes: inflammation severity (0: none; 1: slight; 2: moderate; 3: severe), inflammation range (0: none; 1: mucosal; 2: submucosal; 3: transmural), and crypt damage (0: none; 1: one‐third of the crypt base damage; 2: two‐thirds of the crypt base damage; 3: only surface epithelium is intact; 4: entire crypt and epithelium are missing).

### 2.5 Enzyme‐linked immunosorbent assay (ELIZA)

Mouse interleukin 6 (IL‐6) ELIZA Kit (PI326, Beyotime) and Mouse tumor necrosis factor‐α (TNF‐α) ELIZA Kit (PT512, Beyotime) were used to detect IL‐6 and TNF‐α levels. First, the mouse intestine tissue was homogenized in a homogenizer (SHM1/382) with IGEPAL® CA‐630 buffer (1:5, ST2045, Beyotime), and centrifuged (1000*g*) at 4°C for 10 min. Then, the supernatant for tissues was collected. LPS‐induced macrophages were centrifuged to take the supernatant (500*g* for 5 min).

First, samples were placed in a 96‐well plate. Then, the obtained 100 µL supernatant was added to each well and sealed with sealing film, followed by incubation at room temperature for 2 h. Subsequent to five times of washing with 1× washing buffer, the sample was added with 100 µL biotin conjugate to each well and incubated for 1 h at room temperature with gentle shaking. Post five times of washing again, every well was added with prepared 100 µL streptavidin‐HRP solution, and incubated at room temperature with gentle shaking for 20 min. The solution was discarded, and the sample was washed five times with 1× wash buffer; each well was added with 100 µL 3,3′,5,5′‐tetramethylbenzidine substrate, and incubated at room temperature in the dark with gentle shaking for 20 min. Finally, 50 µL of stop solution was added to each well, and then the absorbance was measured at 450 nm using a microplate reader (Z742711‐1EA, Sigma).

### 2.6 Quantitative reverse transcription polymerase chain reaction (qRT‐PCR)

First, the collected mouse intestine tissue was ground in a mortar with liquid nitrogen, and then transferred to a 1.5 mL centrifuge tube with 1 mL trizol reagent to obtain a homogenate which was left to stand at room temperature for 10 min. After the homogenate was centrifuged (1000*g*) at 4°C for 10 min, the supernatant was collected and extracted by the RNAeasy kit (AM1839, Thermo Fisher Scientific), and the miRNA was extracted by the RNAeasy kit (R0028, Beyotime), followed by detection using microplate reader (Z742711‐1EA, Sigma). Later, cDNA synthesis was performed with reverse transcription kit (D7168L, Beyotime). At last, cDNA, as a template, was amplified by qRT‐PCR instrument (ABI 7500, Thermo Fisher Scientific) in line with the following conditions: predenaturation at 95°C for 10 s, 30 cycles of denaturation at 95°C for 5 s and 60°C for 25 s, and a final elongation step at 70°C for 30 min. By means of the Primer3 Plus (http://www.primer3plus.com/cgi-bin/dev/primer3plus.cgi), the information of primers was obtained: inducible nitric oxide synthase (iNOS): 5′‐GGAGATCAATGGTGTGTC‐3′ (forward) and 5′‐AAGGCAACACCATACC‐3′ (reverse); cyclooxygenase‐2 (COX‐2): 5′‐CATTCTTTGCCCAGCACTTCAC‐3′ (forward) and 5′‐ GACCAGGCACCAGACCAAAGAC‐3′ (reverse); and the reference gene GAPDH: 5′‐CGTGCCGCCTGGAAACCTG‐3′ (forward) and 5′‐AGAGTGGGAGTTGCTGTTGAAGTCG‐3′ (reverse). Relative gene expression was normalized to GAPDH, and calculated according to the $2^{-∆∆C_{t}}$ method.^18^

### 2.7 Western blot

First, mouse intestinal tissue homogenate (with reference to the homogenization step in ELIZA) was obtained. Cells (1 × 10^6^ ~ 1 × 10^7^) were taken from each group and washed with PBS (C0221A, Beyotime), followed by total protein extraction using 0.5 mL of total protein extraction reagent (P0013B, Beyotime). Then, protein concentration was detected by bicinchoninic acid kit (P0011, Beyotime). Proteins and marker (PR1910, 11‐180 kDa, Solarbio) were electrophoresed by SDS‐PAGE (10%, P0690, Beyotime), transferred to PVDF membrane (FFP24, Beyotime), and blocked for 1 h in the blocking solution (P0023B‐100ml, Beyotime) at room temperature.

Next, anti‐p38 (ab31828, 41 kDa, 1:1000, Abcam), antiphosphorylated (p)‐P38 (sc‐166182, 41 kDa, 1:1000, Santa Cruz Biotechnology), anti‐p‐signal‐regulated kinase (ERK) (#9101, 42 kDa, 1:1000, Cell Signaling Scientific, CST, 44 kDa), anti‐ERK (#4695, 42,44 kDa, 1:1000, CST), anti‐Jun N‐terminal kinase (JNK) (ab76572, 48 kDa, 1:5000, Abcam), anti‐p‐JNK (ab124956, 46 kDa, 1:1000, Abcam), anti‐iNOS (ab15323, 140 kDa, 1:250, Abcam), anti‐COX‐2 (ab179800, 69 kDa, 1:1000, Abcam), and anti‐GAPDH (ab8245, 36 kDa, 1:20,000, Abcam) antibodies were added and incubated with the membrane overnight at 4°C. Then, the membrane was removed and washed three times with TBST (P0231, Beyotime) at room temperature.

Finally, HRP‐labeled secondary antibodies (mouse: ab97040, 1:5000; rabbit: ab97080, 1:5000, Abcam) were cultured with the membrane for 1 h at room temperature. Then, the membrane was rinsed off with TBST (P0231, Beyotime), and visualized using ECL detection reagents (P0018S, Beyotime). Protein blots were imaged by a gel imaging system (JS‐1090, peiqing science and technology), and analyzed with Image J (Image J 18.0, National Institutes of Health) analysis software. GAPDH served as an internal reference for calculation of relative expression level.

### 2.8 Cell counting kit‐8 (CCK‐8) assay

The viability of murine macrophage cells was determined using the CCK‐8 kit (C0037, Beyotime) according to the manufacturer's instructions. Cells (2 × 10^3^/well) in 96‐well plates were treated with different concentrations of TEC (0, 1, 10, 50, 100, 200 μM) for 24 h. Next, cells were cultivated with 10 μL of CCK‐8 solution in the dark for 2 h. The absorbance (450 nm) was measured by a microplate reader (Z742711‐1EA, Sigma).

### 2.9 Statistical analysis

**Figure 1:**
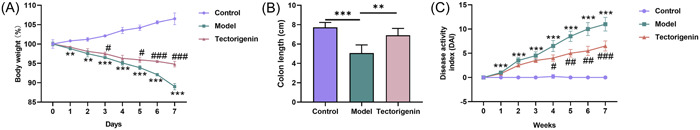
TEC improved DSS‐induced acute colitis in mice. (A) In the C57BL/6 mouse model of IBD, the body weight of each group was measured every day. (B) In the C57BL/6 mouse model of IBD, after euthanasia on the 9th day, the colons of each group were collected and the length was measured. (C) In the C57BL/6 mouse model of IBD, the weight loss rate, diarrhea, and hematochezia of each group were observed and recorded every day to score the disease activity index. ^#^ *p* < .05; ^##^ *p* < .01; ***p < .005; *versus Control; ^#^versus Model. DSS, dextran sodium sulfate; IBD, inflammatory bowel disease; TEC, Tectorigenin.

All measurement data are described by mean ± standard deviation (X ± S). Two‐way analysis of variance (ANOVA) was adopted for analyzing the data in Figure 1A,C. One‐way ANOVA was applied for multigroup comparison, and student's *t*‐test was used for post hoc analyses. Pairwise comparisons between groups were achieved through least significant difference test or Tamhane test. All statistical analyses were realized by GraphPad 8.0 software (GraphPad software Inc.). Statistical significance was indicated when *p* < .05.

## 3 RESULTS

### 3.1 TEC improved DSS‐induced acute colitis in mice

After treatment, on the first 7 days, in the model group compared to the control group, the weight of mice showed a significant downward trend (*p* < .05). In TEC group compared to the model group, the weight of mice was increased (Figure 1A, *p* < .01). As delineated in Figure 1B, after modeling, the colon length of mice was reduced (*p* < .005); however, treatment of TEC increased the colon length (*p* < .01). The DAI scores in Figure 1C reflected that postmodeling, the DAI score of mice was elevated (*p* < .01), but later declined due to the treatment of TEC (*p* < .05). Based on the above information, it can be inferred that TEC can effectively improve DSS‐induced acute colitis in mice.

### 3.2 TEC mitigated DSS‐induced intestinal inflammation

**Figure 2:**
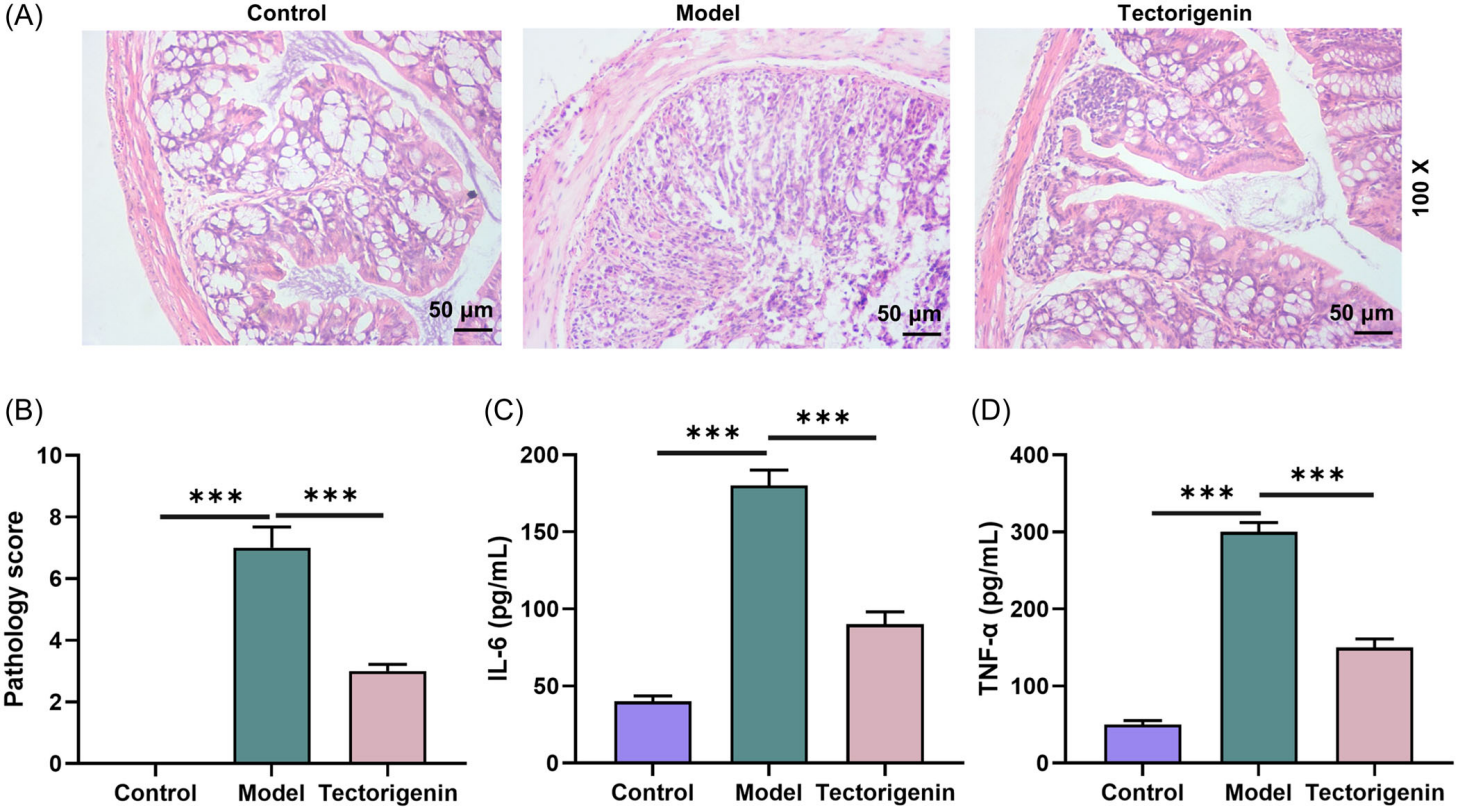
(A and B) After the mice were euthanized on the 9th day, the intestines of the mice in every group were made into colorectal sections and stained with HE. (C) The expression of IL‐6 in mouse intestinal tissue of each group was detected by ELIZA. (D) The expression of TNF‐α in mouse intestinal tissue of each group was measured by ELIZA. ^###^ or ***p < .005; *versus Control; ^#^versus Model. DSS, dextran sodium sulfate; ELIZA, enzyme‐linked immunosorbent assay; HE, hematoxylin and eosin; IL‐6, interleukin 6; TEC, Tectorigenin; TNF‐α, tumor necrosis factor‐α.

HE staining results showed that no obvious lesions were observed in colon tissue of mice in the control group. In the model group, there were apparent inflammatory cell infiltration, incomplete colorectal mucosa and submucosa, local epithelial necrosis and shedding, and a large number of crypts changed or absent. Notably, in TEC group, the colon structure was relatively completed, with a small number of inflammatory cells, gland destruction, and fibrous tissue hyperplasia in the colonic mucosa. The overall symptoms were significantly reduced in TEC group contrasted with model group (*p* < .005, Figure 2A,B).

According to Figure 2B,C, after modeling, IL‐6 and TNF‐α expressions were evidently augmented in model group (*p* < .005), but later diminished by TEC treatment (*p* < .005). All above results proved that TEC can effectively mitigate DSS‐induced inflammation of mouse intestinal tissue.

### 3.3 TEC inhibited expressions of intestinal inflammatory factors and MAPK pathway‐related proteins

**Figure 3:**
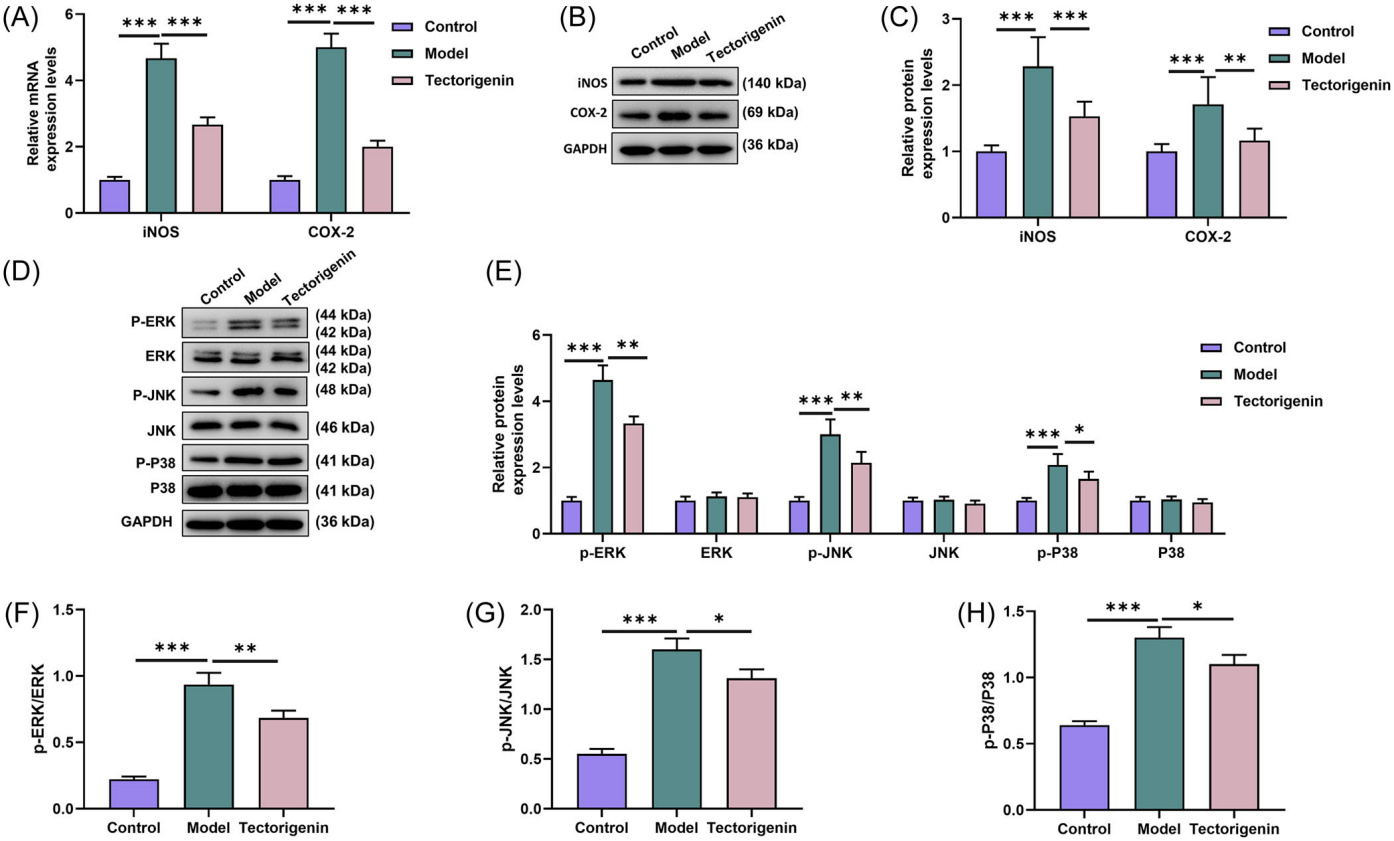
TEC inhibited expressions of intestinal inflammatory factors and MAPK pathway‐related proteins. (A) The mRNA expressions of iNOS and COX‐2 in the mouse intestinal tissue of each group were tested by qRT‐PCR. (B and C) The protein expressions of iNOS and COX‐2 in the mouse intestinal tissue of each group were detected by WB. (D and E) The expressions of MAPK pathway‐related proteins p‐ERK, p‐JNK, and p‐P38 were quantified by WB. (F) p‐ERK/ERK ratio was analyzed in mouse intestinal tissue of each group. (G) p‐JNK/JNK ratio was measured in mouse intestinal tissue of each group. (H) p‐P38/P38 ratio was analyzed in mouse intestinal tissue of each group. ^#^ *p* < .05; ^##^ *p* < .01; ^###^ or ***p < .005; *versus Control; ^#^versus Model. COX‐2, cyclooxygenase‐2; ERK, signal‐regulated kinase; iNOS, inducible nitric oxide synthase; JNK, Jun N‐terminal kinase; MAPK, mitogen‐activated protein kinase; P38, p38 kinase; QRT‐PCR, quantitative reverse transcription polymerase chain reaction; WB, western blot; TEC, Tectorigenin.

The test results of intestinal inflammation‐related factors indicated that iNOS and COX‐2 expression at mRNA and protein levels were increased after modeling (*p* < .005, Figure 3A–C). In model mice, iNOS and COX‐2 expressions were dwindled owing to the treatment of TEC (*p* < .005, Figure 3A–C).

Expressions of MAPK pathway‐related proteins unveiled that relative to the control group, protein expressions of p‐ERK, p‐JNK, and p‐P38 were promoted in model group, while ERK, JNK, and P38 expressions remained unchangeable (*p* < .005, Figure 3D,E). In the TEC group, p‐ERK, p‐JNK, and p‐P38 expression levels were reduced compared to the model group, with unchanged expression levels of ERK, JNK, and P38 (*p* < .005, Figures 3D,E). The ratios of p‐ERK/ERK, p‐JNK/JNK, and p‐P38/P38 were higher in model group than control group (*p* < .005, Figure 3F–H). In the TEC group, the three ratios were reduced compared to the model group (*p* < .05, Figure 3F–H).

The above results mirrored that TEC effectively suppressed DSS‐induced protein and mRNA expressions of inflammation‐related factors and MAPK pathway in mouse intestine.

### 3.4 TEC dwindled expressions of inflammatory factors in LPS‐induced macrophages

**Figure 4:**
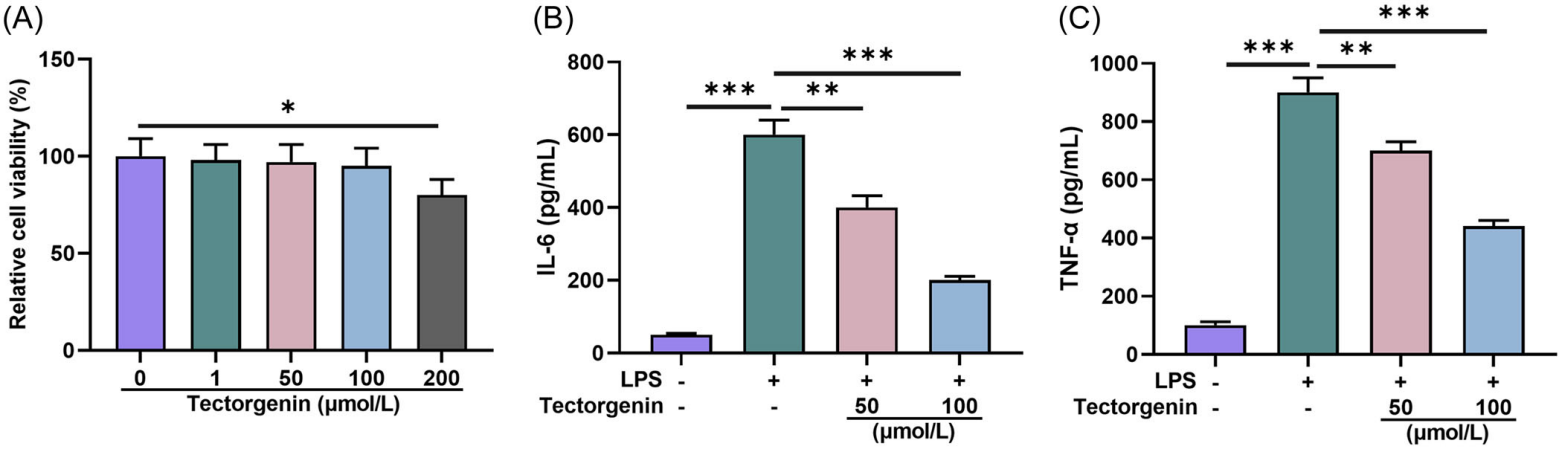
TEC suppressed expressions of inflammatory factors in LPS‐induced macrophages. (A). After macrophages were treated with different concentrations of TEC (0, 1, 10, 50, 100, 200 μM) for 24 h, the viability was tested by CCK‐8. (B). The IL‐6 expression in LPS‐induced macrophages after treatment of 50 and 100 μM TEC was detected by ELIZA. (C). The TNF‐α expression in LPS‐induced macrophages after treatment of 50 and 100 μM TEC was quantitated by ELIZA. *p < .05; ^##^ *p* < .01; ^###^ or ***p < .005; *versus Control; ^#^versus LPS. CCK‐8, cell counting kit‐8; ELIZA, enzyme‐linked immunosorbent assay; IL‐6, interleukin 6; LPS, lipopolysaccharide; TEC, Tectorigenin; TNF‐α, tumor necrosis factor‐α.

When cells were treated with TEC of 200 μM, cell viability was significantly weakened (*p* < .05, Figure 4A). Based on the results of related research, 50 and 100 μM TEC was selected for subsequent experiments.

The expression levels of inflammatory factors IL‐6 and TNF‐α were increased in the LPS group relative to the control group (*p* < .005, Figure 4B,C); while levels of these factors were decreased in TEC 50/100 μM group relative to LPS group (*p* < .005, Figure 4B,C).

The above results proved that TEC (50 and 100 μM) can attenuate the inflammation of LPS‐induced macrophages.

### 3.5 TEC downregulated expressions of inflammatory factors and MAPK pathway‐related proteins in LPS‐induced macrophages

**Figure 5:**
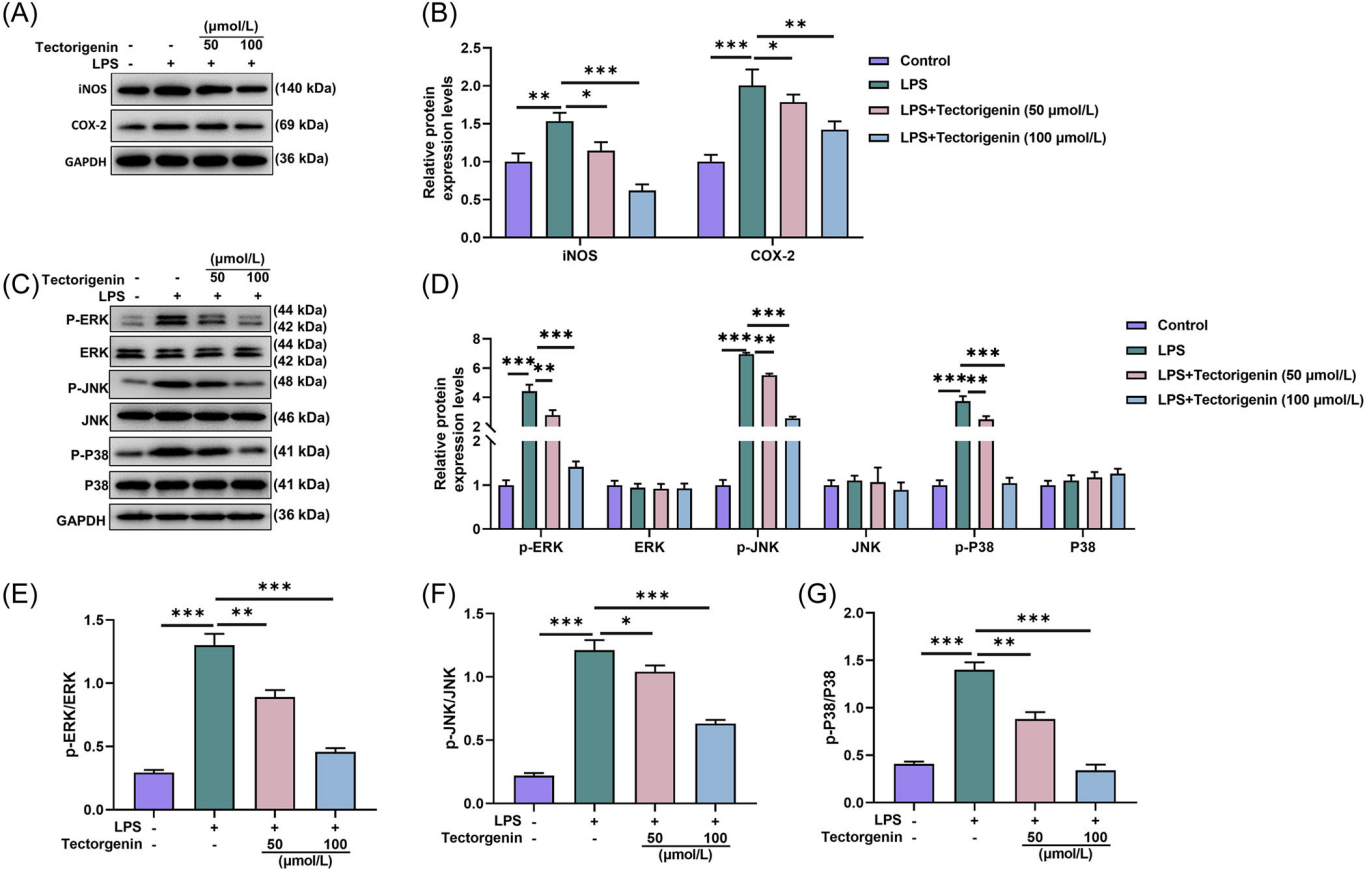
TEC downregulated expressions of inflammatory factors and MAPK pathway‐related proteins in LPS‐induced macrophages. (A and C) The protein and mRNA expressions of iNOS and COX‐2 were detected by WB and qRT‐PCR in LPS‐induced macrophages. (D and E) The expressions of MAPK pathway‐related proteins p‐ERK, p‐JNK, and p‐P38 were quantified by WB in LPS‐induced macrophages. (F) p‐ERK/ERK ratio was analyzed in LPS‐induced macrophages. (G) p‐JNK/JNK ratio was detected in LPS‐induced macrophages. (H) p‐P38/P38 ratio was determined in LPS‐induced macrophages. ^#^ *p* < .05; ** or ^##^ *p* < .01; ^###^ or ***p < .005; *versus Control; ^#^versus LPS. COX‐2, cyclooxygenase‐2; ERK, signal‐regulated kinase; iNOS, inducible nitric oxide synthase; JNK, Jun N‐terminal kinase; LPS, lipopolysaccharide; MAPK, mitogen‐activated protein kinase; P38, p38 kinase; QRT‐PCR, quantitative reverse transcription polymerase chain reaction; TEC, Tectorigenin; WB, western blot.

As described in Figure 5A,B, relative to the control group, iNOS and COX‐2 protein expression levels were increased following LPS induction (*p* < .01). Through treatment of TEC (50/100 μM), IL‐6 and TNF‐α protein expressions were reduced in LPS‐induced cells (*p* < .05, Figure 5A,B).

The detection results of MAPK pathway‐related protein expressions unveiled that LPS induction elevated p‐ERK, p‐JNK, and p‐P38 protein expression levels in cells (*p* < .005, Figure 5C,D), which was later lessened by TEC (50/100 μM) (*p* < .01, Figure 5C,D). Besides, LPS induction increased the ratios of p‐ERK/ERK, p‐JNK/JNK, and p‐P38/P38 in cells (*p* < .005, Figure 5E–G), and TEC (50/100 μM) also suppressed these upregulation trends caused by LPS (*p* < .05, Figure 5E–G).

These data demonstrated that TEC at 50 and 100 μM partially inhibited expressions of inflammatory factors and MAPK pathway‐related proteins in LPS‐induced macrophages.

## 4 DISCUSSION

IBD refers to idiopathic intestinal inflammatory diseases that involve rectum and colon.^19^ DSS could destroy the structure of the intestine, causing colorectal inflammatory disease, weight loss, bleeding, and colon shortening.^13^, ^20^ Therefore, this study used the DSS‐induced IBD model mice to histologically evaluate the efficacy of TEC. In the model mice, it was found that injection with TEC continuously improved the IBD, as evidenced by increased mouse body weight, lower DAI score, the suppressed shortening of colon length and the alleviation of colon histopathological changes, revealing the therapeutic effect of TEC on IBD.

To further explore the specific mechanism of TEC in treating IBD, we also detected the changes of MAPK signaling pathway and inflammatory factors. The MAPK pathway, which has three subunits of ERK, JNK, and P38, has been confirmed to be activated in the IBD model.^21^ The MAPK pathway mediates inflammation through phosphorylating ERK, JNK, and P38 into p‐ERK, p‐JNK, and p‐P38.^22^ A related study has found that Andrographis improves inflammation by inhibiting the expressions of p‐ERK, p‐JNK, and p‐P38.^15^ In mice with fulminant hepatitis, TEC protects the liver from inflammatory damage via reducing the expressions of TLR4, p‐ERK, p‐JNK, and p‐P38.^17^ Similarly, this study found that MAPK pathway was activated in the intestinal tissue of IBD mice, while TEC inhibited the expressions of p‐ERK, p‐JNK, and p‐P38. The phosphorylation of MAPK‐related signaling molecules have been reported to induce the release of many proinflammatory mediators such as IL‐6, COX‐2, iNOS, and TNF‐α, in microglia.^23^ In the IBD model, the important roles of IL‐6, TNF‐α, iNOS, and COX‐2 have been identified in inflammation.^24^, ^25^ The obvious expression of iNOS is accompanied by upregulated COX‐2 in the inflammation site, which leads to aggravated inflammation.^26^ During inflammation, the increased expression levels of TNF‐α, IL‐6, iNOS, and COX‐2 can be observed at the inflammation site.^27^ In mice with fulminant hepatitis, TEC can protect the liver against inflammatory damage by downregulating TLR4, IL‐6, TNF‐α, iNOS, and COX‐2 expressions.^21^ Consistent with previous studies, this study unraveled that TEC inhibited the activation of inflammatory pathways, that is, IL‐6, TNF‐α, iNOS, and COX‐2 protein expressions were inhibited, which illustrated that TEC can effectively improve the inflammatory response of mouse intestinal tissue induced by DSS. In conclusion, the findings of in vivo experiments showed that TEC mitigated the enteritis tissue inflammatory response of DSS‐induced mouse via inhibiting the activation of MAPK pathway. However, the specific molecular mechanism needs to be studied through in vitro experiments, and thus further exploration was carried out on macrophages in this study.

The functions of macrophage include the production of proinflammatory mediators and the enhancement of inflammatory response, leading to many inflammatory diseases, including IBD.^28^ LPS has been verified to induce macrophage inflammation and activate the MAPK signaling pathway.^29^ Therefore, the LPS‐induced macrophage inflammation model can be used to evaluate the anti‐inflammatory effects of various agents.^30^ On these grounds, this study established an LPS‐induced macrophage model to evaluate the effect of TEC on the MAPK pathway in the inflammatory response. A former report found that the expressions of p‐ERK, p‐JNK, and p‐P38 of MAPK pathway are promoted in LPS‐induced macrophages.^31^ Also, LPS activates the expressions of inflammatory factors IL‐6, TNF‐α, iNOS, and COX‐2 in macrophage.^16^, ^32^ In the current study, we confirmed that TEC at 50 and 100 μM inhibited the expressions of proinflammatory factors (IL‐6, TNF‐α, iNOS, and COX‐2) and MAPK pathway‐related proteins (p‐ERK, p‐JNK, p‐P38), implying that TEC can block the activation of MAPK pathway to alleviate the inflammatory response of LPS‐treated macrophages.

In summary, the results of in vivo and in vitro experiments are consistent. This study proved that TEC effectively improves IBD in mice and the inflammation of LPS‐induced macrophages via inhibiting the MAPK signaling pathway, manifesting the potential of TEC as a treatment drug for IBD. However, the specific therapeutic regime needs to be formulated based on considerable animal experiments and human experiments, and other possible pathways being modulated will be further explored in the future. In addition, TEC has a great reference value for other diseases with the same inflammatory pathway, which is worthy of further exploration.

## AUTHOR CONTRIBUTIONS

Hong Huang provide substantial contributions to conception and design. Sanhui Tang, Yanghong Zhou, and Yi Cai contribute data acquisition, data analysis, and interpretation. Hong Huang drafting the article or critically revising it for important intellectual content. Hong Huang, Sanhui Tang, Yanghong Zhou, and Yi Cai final approval of the version to be published. Hong Huang, Sanhui Tang, Yanghong Zhou, and Yi Cai agreement to be accountable for all aspects of the work in ensuring that questions related to the accuracy or integrity of the work are appropriately investigated and resolved.

## CONFLICT OF INTEREST STATEMENT

The authors declare no conflict of interest.

## ETHICS STATEMENT

All animal experimental procedures were approved by the Committee of Zhejiang Baiyue Biotech Co., Ltd. for Experimental Animals Welfare (approval number: ZJBYLA‐IACUC‐20230107) and performed in accordance with the guidelines of the National Institutes of Health on Animal Care and Use.

## Data Availability

The analyzed data sets generated during the study are available from the corresponding author on reasonable request.
